# Effectiveness of arginine vasopressin for the management of refractory hemorrhagic shock in a patient with autonomic dysreflexia caused by spinal cord injury

**DOI:** 10.1186/s40981-018-0216-8

**Published:** 2018-11-12

**Authors:** Tsukasa Shimauchi, Jun Maki, Jun Yoshino, Naoyuki Fujimura, Sumio Hoka

**Affiliations:** 1 Operating Rooms, Kyushu University Hospital, 3-1-1 Maidashi, Higashi-ku, Fukuoka, 812-8582 Japan; 2 Emergency and Critical Care Center, Kyushu University Hospital, Fukuoka, Japan; 3 Department of Anesthesiology, St Mary’s Hospital, Kurume, Japan; 4 Department of Anesthesiology and Critical Care Medicine, Kyushu University Graduate School of Medical Sciences, Fukuoka, Japan

**Keywords:** AVP, Arginine vasopressin, Hemorrhagic shock, Autonomic dysreflexia, Spinal cord injury

## Abstract

**Background:**

Arginine vasopressin has been used for the management of refractory vasodilatory shock. However, it is still unclear whether arginine vasopressin is useful for hypotension in patients with spinal cord injury.

**Case description:**

A 78-year-old man with autonomic dysreflexia and paralysis below the level corresponding to Th2 due to spinal cord injury previously underwent cholecystectomy. During the surgery, accidental hemorrhage led him to refractory hemorrhagic shock unresponsive to fluid resuscitation and catecholamine. Lasting hypotension was improved with arginine vasopressin.

**Conclusion:**

We described a rare case report on the use of arginine vasopressin for management of refractory hemorrhagic shock in a patient with autonomic dysreflexia.

## Background

Prolonged hypotension due to marked hemorrhage often progresses to shock that is unresponsive to fluid resuscitation and catecholamines such as norepinephrine [1–3]. Several reports have described the usefulness of arginine vasopressin (AVP) for hemorrhagic shock unresponsive to volume replacement and drug intervention [3–7]. However, there are no reports on the use of AVP for the refractory hemorrhagic shock in patients with autonomic dysreflexia caused by spinal cord injury. Autonomic dysreflexia is a cardiovascular disorder and direct cause of death in patients with spinal cord injury [8, 9]. It manifests in both the acute and chronic stages of spinal cord injury and becomes refractory in complete injury above the Th6 level [8]. Autonomic dysreflexia leads to cardiac dysfunction because of hyperresponsiveness of overexpression of catecholamine receptors [9, 10].

Here, we describe a case of catecholamine-resistant hemorrhagic shock treated by AVP in a patient with autonomic dysreflexia caused by spinal cord injury.

## Case description

**Fig. 1:**
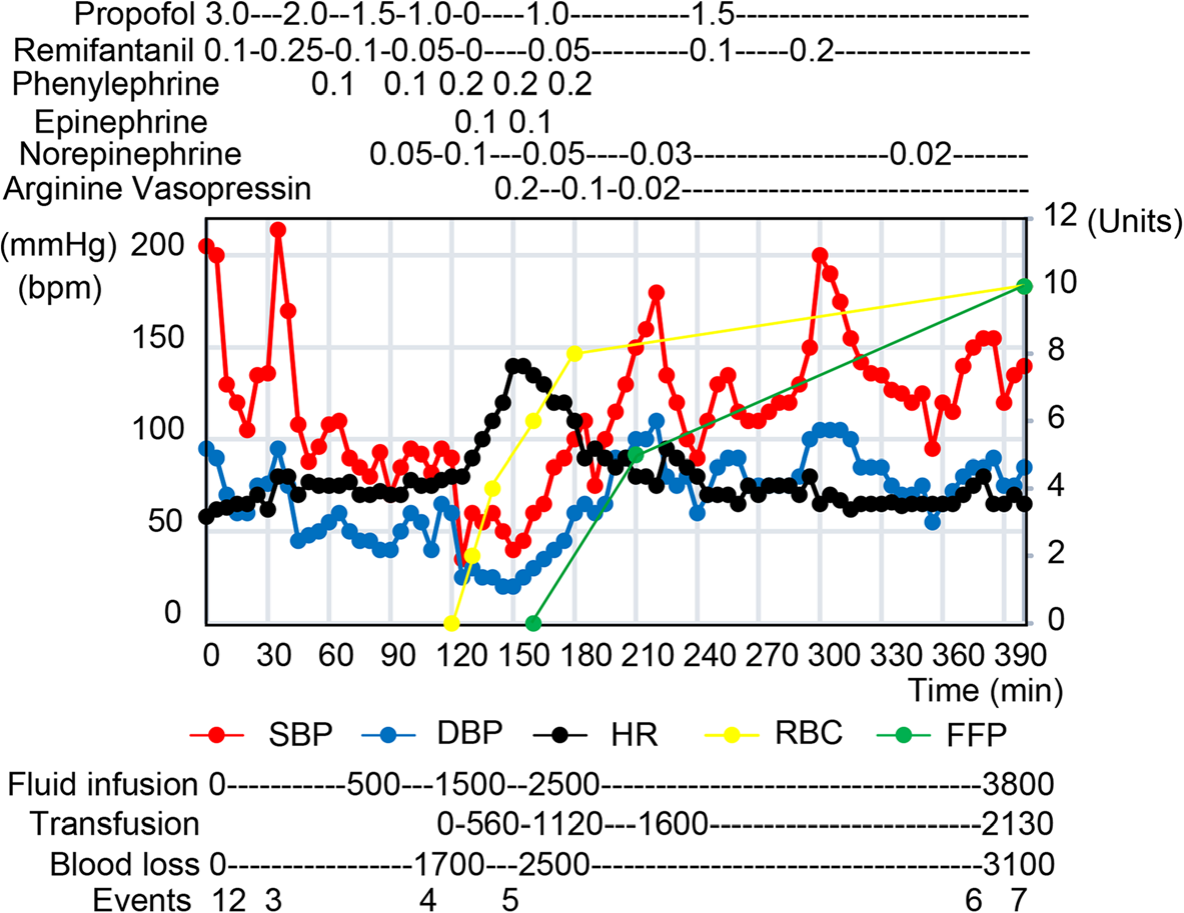
Anesthetic records for the patient. Propofol: target controlled infusion (μg/ml). Remifentanil: continuous infusion (μg/kg/min). Phenylephrine: bolus injection (mg), Epinephrine: bolus injection (mg). Norepinephrine: continuous injection (μg/kg/min), Arginine vasopressin: continuous injection (U/kg/h). Fluid infusion, transfusion, and blood loss are represented as milliliter. 1, start of anesthesia. 2, tracheal intubation. 3, start of surgery. 4, injury to and 5, repair of the inferior vena cava. 6, end of surgery. 7, end of anesthesia

Written informed consent was obtained from the patient for publication of this case report. A 78-year-old man who had recurrent cholecystolithiasis was scheduled for cholecystectomy. He was paralyzed below the Th2 level due to spinal cord injury caused by traffic accident at the age of 45. He had clinical features of autonomic dysreflexia such as hypertension, headache, sweating, flushing, and loss of consciousness due to hypotension in the sitting position. Preoperative examination revealed no other abnormalities, and laboratory data were unremarkable. General anesthesia was induced with fentanyl, propofol, and rocuronium, and maintained with propofol, remifentanil, and air-mixed oxygen. Arterial pressure was monitored directly from the radial artery. The systolic and mean arterial pressures (SBP and MAP) were maintained around 100 and 75 mmHg, respectively, and the heart rate (HR) was 75 bpm. During manual dissection of the adhesions around the cystic duct, the inferior vena cava was accidentally injured and marked hemorrhage occurred and then blood pressure had decreased abruptly. Blood loss was replaced with 1500 ml of crystalloid before transfusion of red blood cells and fresh frozen plasma. Phenylephrine and epinephrine bolus, and continuous infusion of norepinephrine (0.1 μg/kg/min) were performed in order to maintain systolic blood pressure > 70 mmHg, a threshold for increased mortality in patients with hemorrhagic shock [11]. Although the inferior vena cava was surgically repaired and hemostasis was almost restored, hypotension (systolic blood pressure 40 mmHg) persisted. Blood gas data were pH 7.19, PaO2 272 mmHg, bicarbonate 11.9 mEq/l, base deficit − 15 mEq/l, and hemoglobin 6.1 g/dL (10.1 g/dL before surgery). Thus, we decided to administer AVP and started continuous infusion at 0.2 U/kg/min, and the hemodynamic status immediately improved. We titrated the infusion rate to 0.02 U/kg/h (Fig. 1). 80 mEq of sodium bicarbonate was administered, and fluid resuscitation was continued. The SBP and MAP increased to 110 and 85 mmHg respectively, and the HR was 90 bpm. His arterial blood pressure was maintained with 0.02 U/kg/h of AVP and 0.02 μg/kg/min of norepinephrine at the end of the operation. The ABG at this time demonstrated a pH of 7.43, Pco_2_ of 37 mmHg, Pao_2_ of 194 mmHg, bicarbonate of 17.6, a base deficit of − 7, and Hb of 8.5 g/dL. The total blood loss was 3100 ml. For the resuscitation from hypovolemic shock, 2130 ml of blood components was transfused and 3800 ml of crystalloid was administered. The patient was admitted to the intensive care unit with intubated. He had no prolonged hypotension and no neurological complication after surgery.

## Discussion

For the management of patients with hemorrhage shock, volume resuscitation to maintain adequate tissue perfusion takes priority [12]. However, in cases of refractory hemorrhage shock, prolonged hypotension leads to hypoperfusion of the brain and other vital organs. Although vasopressors, such as norepinephrine and dopamine, are useful for maintaining arterial pressure [12], our patient was in a state of hemorrhage shock that was unresponsive to volume resuscitation and catecholamines. This is the first case report describing the use of AVP for the management of refractory hemorrhagic shock in a patient with autonomic dysreflexia caused by spinal cord injury.

Hemorrhagic shock leads to the immediate release of catecholamines, such as epinephrine and norepinephrine, and delayed activation of the renin-angiotensin system as compensatory responses [12]. These compensatory systems may be poorly activated in patients with autonomic dysreflexia. Acidosis followed by hemorrhagic shock inactivates catecholamine receptors and autonomic dysreflexia downregulates catecholamine receptors [8]. Furthermore, prolonged hemorrhagic shock causes vasodilation via the production of nitric oxide (NO) and depletion of stored AVP [7, 12, 13]. Advanced hemorrhagic shock becomes unresponsive to both volume resuscitation and catecholamines because of vasodilatation and acidosis [7, 12]. In cases of hemorrhagic shock unresponsive to catecholamines, vasoconstriction with AVP is an excellent strategy to avoid brain and organ ischemia.

We first administered AVP at 0.2 U/kg/h in this case even though this dose is higher than in previous reports [3, 4]. However, there are case reports that describe high-dose AVP for patients with severe hemorrhagic shock [14]. Therefore, we consider 0.2 U/kg/h of AVP to be appropriate for patients with severe hemorrhagic shock.

AVP restores vascular tone in a catecholamine-resistant shock state via four known mechanisms: activation of V_1_ receptors, subtype of AVP receptor found on vascular smooth muscle cells, modulation of ATP sensitive K^+^ channels, inhibitory action on NO, and potentiation of adrenergic and other vasoconstrictor agents [15]. V_1_ receptors are highly expressed in vascular smooth muscle and cause vasoconstriction though an increase in intracellular calcium via phosphatidylinositol-bisphosphonate [15]. Inhibition of NO is effective for hemorrhagic shock that causes vasodilation via NO release [12]. AVP increases systemic vascular resistance without stimulating catecholamine receptors and has no impact on heart rate. AVP also minimally affects pulmonary vascular resistance [16]. For these reasons, AVP is more beneficial than catecholamine for patients with impaired cardiac function [16].

## Conclusion

AVP may be useful for the management of marked hemorrhagic shock that is unresponsive to volume replacement and catecholamines, especially for patients with autonomic dysreflexia caused by spinal cord injury.

## Abbreviations

AVP: Arginine vasopressin
HR: Heart rate
MAP: Mean arterial pressure
NO: Nitric oxide
SBP: Systolic arterial pressure
